# Supplementary material for: Separation of α-Lactalbumin-Enriched Fractions from Caprine and Ovine Native Whey Concentrate by Combining Membrane and High-Pressure Processing
Source: Foods. 2023 Jul 12;12(14):2688. doi: 10.3390/foods12142688 (PMC10378926; doi:10.3390/foods12142688)
Supplement: Supplementary file 1 [file foods-12-02688-s001.zip › Figure S1.pdf]

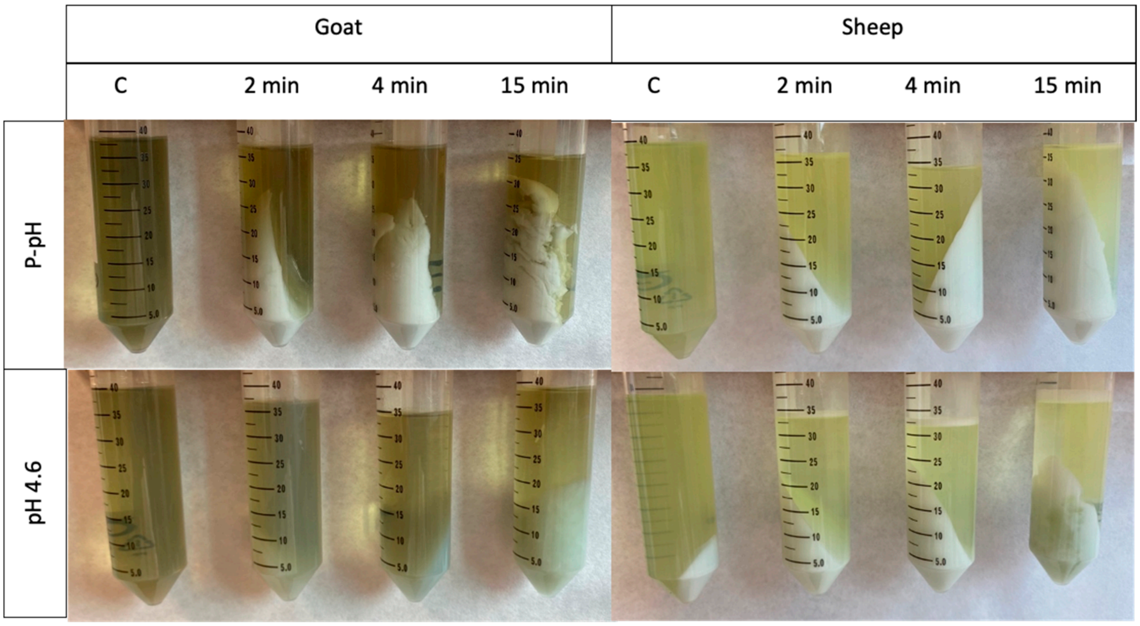

Figure S1. Visual appearance of caprine (left) and ovine (right) NWC_{sup} after HPP (600 MPa, 23 °C) at physiological (P-pH) and 4.6 pH for 0 (control) 2, 4 and 15 min.
