# Supplementary material for: Separation of α-Lactalbumin-Enriched Fractions from Caprine and Ovine Native Whey Concentrate by Combining Membrane and High-Pressure Processing
Source: Foods. 2023 Jul 12;12(14):2688. doi: 10.3390/foods12142688 (PMC10378926; doi:10.3390/foods12142688)
Supplement: Supplementary file 1 [file foods-12-02688-s001.zip › Table S1.pdf]

Table S1. Protein concentrations and main process performance parameters of α-Lactalbumin (α-La) enriched fraction after high-pressure processing (HPP) of ovine native whey concentrate (NWC) at 600 MPa at 23ºC (supernatant). Each value is expressed as mean value ± SD (n = 3). Different small letters in the same column indicate significant ($P < 0.05$) differences according to Tukey test. *Significant differences regarding the control (untreated) NWC (T-Test).

pH	HPP processing (min)	a-La concentration (mg/mL)	b-Lg B concentration (mg/mL)	b-Lg A concentration (mg/mL)	b-Lg A + B concentration (mg/mL)	Y_{a-La} (%)	Pur_{a-La} (%)	$Pre_{b-Lg\ B}$ (%)	$Pre_{b-Lg\ A}$ (%)	$Pre_{b-Lg\ A+B}$ (%)
P-pH	**Control** (untreated)	32.35 ± 1.65^{cd}	51.89 ± 2.19^{b}	54.56 ± 2.19^{a}	106.46 ± 4.37^{b}	-	23.30 ± 0.19^{e}	-	-	-
	2	34.04 ± 0.54^{bc}	3.37 ± 0.46^{d}	2.97 ± 0.51^{d}	6.33 ± 0.97^{de}	62.01 ± 2.17^{b*}	84.35 ± 1.83^{b}	96.21 ± 0.58^{a*}	96.83 ± 0.61^{a*}	96.53 ± 0.60^{ab*}
	4	29.09 ± 2.23^{d}	2.34 ± 0.10^{d}	1.78 ± 0.16^{d}	4.12 ± 0.13^{e}	44.83 ± 4.60^{c*}	87.56 ± 0.95^{ab}	97.78 ± 0.15^{a*}	98.40 ± 0.14^{a*}	98.10 ± 0.10^{a*}
	15	15.29 ± 0.08^{e}	0.52 ± 0.03^{d}	0.55 ± 0.01^{d}	1.03 ± 0.05^{e}	20.71 ± 1.66^{d*}	93.68 ± 0.26^{a}	99.58 ± 0.05^{a*}	99.59 ± 0.05^{a*}	90.58 ± 0.05^{a*}
4.6	**Acidified** (untreated)	37.78 ± 0.50^{ab}	65.55± 0.57^{a}	59.40 ± 0.59^{a}	124.95 ± 1.13^{a}	114.14 ± 1.03^{a*}	23.21 ± 0.16^{e}	-22.53 ± 1.07^{c*}	-5.37 ± 1.04^{d}	-13.72 ± 1.03^{d*}
	2	38.44 ± 1.00^{a}	30.62 ± 8.50^{c}	31.18 ± 5.34^{b}	61.80 ± 13.73^{c}	62.76 ± 6.08^{b*}	38.84 ± 5.61^{d}	68.98 ± 9.57^{b*}	70.02 ± 6.67^{c*}	69.52 ± 7.98^{c*}
	4	38.38 ± 1.03^{ab}	25.64 ± 2.40^{c}	26.86 ± 1.45^{b}	52.49 ± 3.81^{c}	69.06 ± 5.79^{b*}	42.27 ± 1.32^{d}	71.54 ± 3.16^{b*}	71.70 ± 2.40^{c*}	71.62 ± 2.73^{c*}
	15	32.85 ± 2.96^{cd}	8.95 ± 0.55^{d}	11.26 ± 1.71^{c}	20.21 ± 2.26^{d}	62.14 ± 5.45^{b*}	61.94 ± 1.65^{c}	89.54 ± 0.86^{a*}	87.50 ± 2.07^{b*}	88.49 ± 1.50^{b*}

(Y_{a-La}) = a-La yield. (Pur_{a-La}) = a-La Purification degree. (Pre_{b-LgA}) = b-Lg A precipitation degree. (Pre_{b-LgB}) = b-Lg B precipitation degree.
