# Supplementary material for: Separation of α-Lactalbumin-Enriched Fractions from Caprine and Ovine Native Whey Concentrate by Combining Membrane and High-Pressure Processing
Source: Foods. 2023 Jul 12;12(14):2688. doi: 10.3390/foods12142688 (PMC10378926; doi:10.3390/foods12142688)
Supplement: Supplementary file 1 [file foods-12-02688-s001.zip › Table S2.pdf]

Table S2. Protein concentrations and main process performance parameters of α-Lactalbumin (α-La) enriched fraction after high-pressure processing (HPP) of caprine native whey concentrate (NWC) at 600 MPa at 23ºC (supernatant). Each value is expressed as mean value ± SD (n = 3). Different small letters in the same column indicate significant ($P < 0.05$) differences according to Tukey test. *Significant differences regarding the control (untreated) NWC (T-Test).

pH	HPP processing (min)	a-La concentration (mg/mL)	b-Lg concentration (mg/mL)	$Y_{a\text{-}La}$ (%)	$Pur_{a\text{-}La}$ (%)	$Pre_{b\text{-}Lg}$ (%)
P-pH	**Control** (untreated)	21.24 ± 1.65^{b}	81.94 ± 4.84^{b}	-	20.57 ± 0.30^{e}	-
	2	27.93 ± 0.53^{a}	6.91 ± 0.89^{de}	75.61 ± 4.12^{cd*}	80.21 ± 2.08^{b}	95.16 ± 0.70^{a*}
	4	25.49 ± 0.42^{a}	3.02 ± 0.69^{e}	64.52 ± 5.68^{de*}	89.45 ± 2.06^{a}	97.99 ± 0.63^{a*}
	15	17.87 ± 3.18^{b}	1.39 ± 0.01^{e}	30.22 ± 7.73^{f*}	96.85 ± 0.62^{a}	99.39 ± 0.08^{a*}
4.6	**Acidified** (untreated)	26.79 ± 0.12^{a}	103.61 ± 1.03^{a}	129.86 ± 0.56^{a*}	20.54 ± 0.14^{e}	-30.16 ± 1.30^{d*}
	2	25.86 ± 1.10^{a}	80.11 ± 3.59^{b}	94.70 ± 7.05^{b}	24.41 ± 0.65^{de}	23.82 ± 8.10^{c*}
	4	26.96 ± 1.04^{a}	62.42 ± 11.61^{c}	84.62 ± 3.98^{bc}	30.49 ± 3.44^{d}	49.23 ± 9.54^{b*}
	15	25.56 ± 0.97^{a}	17.86 ± 3.01^{d}	56.22 ± 10.87^{e*}	59.06 ± 3.89^{c}	89.93 ± 1.88^{a*}

($Y_{a\text{-}La}$) = a-La yield. ($Pur_{a\text{-}La}$) = a-La Purification degree. ($Pre_{b\text{-}LgA}$) = b-Lg A precipitation degree. ($Pre_{b\text{-}LgB}$) = b-Lg B precipitation degree.
